# Deep learning approach for an interface structure analysis with a large statistical noise in neutron reflectometry

**DOI:** 10.1038/s41598-021-02085-6

**Published:** 2021-11-22

**Authors:** Hiroyuki Aoki, Yuwei Liu, Takashi Yamashita

**Affiliations:** 1 Materials and Life Science Division, J-PARC Center, Japan Atomic Energy Agency, 2-4, Shirakata, Tokai, Ibaraki 319-1195 Japan; 2 Institute of Materials Structure Science, High Energy Accelerator Research Organization, 203-1, Shirakata, Tokai, Ibaraki 319-1106 Japan; 3 AdvanceSoft, Corp., 4-3, Kandasurugadai, Chiyoda, Tokyo, 101-0062 Japan

**Keywords:** Nanometrology, Characterization and analytical techniques, Polymers

## Abstract

Neutron reflectometry (NR) allows us to probe into the structure of the surfaces and interfaces of various materials such as soft matters and magnetic thin films with a contrast mechanism dependent on isotopic and magnetic states. The neutron beam flux is relatively low compared to that of other sources such as synchrotron radiation; therefore, there has been a strong limitation in the time-resolved measurement and further advanced experiments such as surface imaging. This study aims at the development of a methodology to enable the structural analysis by the NR data with a large statistical error acquired in a short measurement time. The neural network-based method predicts the true NR profile from the data with a 20-fold lower signal compared to that obtained under the conventional measurement condition. This indicates that the acquisition time in the NR measurement can be reduced by more than one order of magnitude. The current method will help achieve remarkable improvement in temporally and spatially resolved NR methods to gain further insight into the surface and interfaces of materials.

## Introduction

Neutron reflectometry (NR) has been used extensively to discuss the spatial distribution of the atomic composition in the depth direction^1–3^ for the structure analysis of the surface and interfaces of materials. The surface and interface structure of a sample can be characterized as the depth distribution of the neutron scattering length density (SLD), which is determined by the isotopic and spin state for the constituent atoms of the sample via the analysis of the NR profile. The NR measurement performed using isotope-labeled samples (e.g., the substitution of $^1$H to $^2$H in a molecular structure) enables the selective characterization of the labeled part. Further, spin-polarized neutrons allow the characterization of the magnetic structure at the interfaces. NR provides structural information about the surfaces and interfaces of a specimen that cannot be obtained using other techniques; therefore, it is employed for analyzing the surfaces, interfaces, and thin films in various research fields such as magnetism^4–6^ and soft matters^7–11^. A complementary measurement of NR with the other methods having different contrast mechanisms such as X-ray reflectometry and ellipsometry provides the further detailed information on the surface/interface structure of the materials. However, the flux of the neutron beam is low compared to that of the other sources such as synchrotron radiation. A measurement time on the order of hours is required even at nuclear reactors and accelerator facilities providing the highest intensity neutron beams to obtain an NR profile with a sufficiently low statistical error for the data analysis to determine an SLD distribution with a sub-nanometric accuracy. Such a relatively long measurement time places a limitation on the use of NR for analyzing the time-dependent structure dynamics^11–13^; therefore, the data acquisition time needs to be shortened without compromising the resolution for the structure characterization to improve the temporal resolution of the time-dependent measurement. On the other hand, novel NR imaging techniques combined with computed tomography have been developed recently to enable the structural analysis of surfaces and interfaces with in-plane inhomogeneity, which cannot be conducted by the conventional NR^14,15^. However, such the analysis requires a long measurement time on the scale of days because of the acquisition of a large number of data with rotating a sample. The substantial decrease in the data acquisition time will overcome the restrictions in the conventional NR and provide further insight into various phenomena related to surfaces and interfaces. This can be achieved by increasing the neutron beam power; however, another approach should be developed because of the difficulties in the construction of a huge neutron source facility.

This study aims at the extraction of the true NR profile hidden in a large statistical noise to enable the reliable data analysis with the experimentally obtained data in a short acquisition time. Signal restoration from signals and images including large noise has been extensively studied for various purposes, and machine learning techniques have been widely used for signal processing in various applications^16,17^. Previous studies showed that deep learning methods with convolutional neural network (CNN) models are highly effective for decreasing the noise from digital signals and images^18–23^; therefore, a similar approach would also work for the NR experiments. A few applications of the machine learning in NR have been intended to directly determine the structure of the surface and interface of a specimen^24–26^. The current study introduces a deep learning approach that uses neural networks to restore the reflection profile hidden in the large statistical noise to overcome the limitation in the conventional NR methods. The signal restoration in NR using a digital filter and neural networks is discussed. We demonstrate the SLD distribution analysis for the NR data with a weaker signal intensity by an order of magnitude compared to that for the conventional data.

## Methods

Supervised learning was performed using the data set of the calculated ground truth reflection intensity profiles and the simulated NR experiment data considering the measurement condition at a neutron reflectometer SHARAKU installed at the Materials and Life Science Experiment Facility (MLF) in the Japan Particle Accelerator Research Complex (J-PARC)^27,28^. In a typical experiment at SHARAKU, a neutron reflectivity spectrum is obtained using an incident neutron beam with a wavelength range of 0.22–0.88 nm at angles of 0.3$^\circ$, 0.7$^\circ$, 1.6$^\circ$, and 3.5$^\circ$; this covers the momentum transfer range of 0.075–0.30, 0.17–0.70, 0.40–1.6, and 0.87–3.5 $\hbox {nm}^{-1}$, respectively. A number of 50,000 neutrons are acquired are at the incident angle of 0.3$^\circ$ and 20,000 neutrons are acquired at the other angles. We define this measurement condition as the “standard” condition for the NR measurement. For the training data, the ground truth NR data were calculated for randomly generated $2.4 \times 10^5$ sample structures at the incidence angles used in the experiment at SHARAKU. For a given sample structure, the ground truth NR is theoretically calculated by the Parratt’s formulation^29^. A given numbers of neutron events was generated according to the Poisson statistics to reproduce the theoretical neutron reflection intensity spectra calculated by the reflectivity and incident neutron spectrum observed at SHARAKU. For each structure, the data with the total neutron numbers of 10%, 5%, 2.5%, and 1.5% of the standard condition were generated, resulting in the training data sets of $9.6 \times 10^5$. The details on the generation procedure of the simulated NR data are described in the Supplementary Information. Supervised training using the data set of the simulated NR profiles and corresponding ground truth was conducted using Pytorch on a workstation equipped with two GPU boards (GeForce RTX3090, NVIDIA). The detailed information is available in the Supplementary Information. In the training, 80% and 20% of the data sets were used for the training and the validation, respectively.

The NR measurement was conducted for thin films of poly(methyl methacrylate) (PMMA) and poly(vinyl alcohol) (PVA) at SHARAKU to apply the obtained deep learning based data processing for the experimental data. The accelerator operation power was 600–700 kW. The experimental details are described in the Supplementary Information. The NR data was analyzed by a reflectometry analysis software package, *refnx*^30^.

## Results and discussion

**Figure 1:**
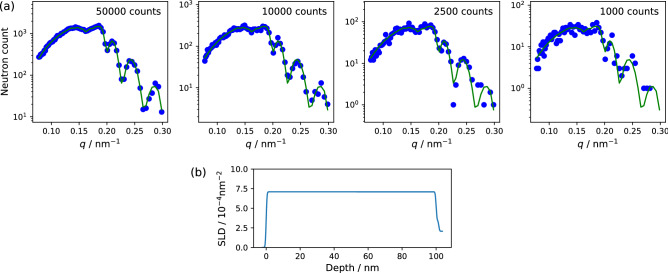
Simulated NR data at the incidence angle of 0.3$^\circ$ with the total neutron numbers of 50,000, 10,000, 2500, and 1000 (**a**) and depth distribution of SLD (**b**) for a 100-nm-thick film with SLD of $7.1 \times 10^{-4}$ $\hbox {nm}^{-2}$ . The blue filled circles and green curves in (**a**) represent the simulated NR and ground truth data, respectively.

**Figure 2:**
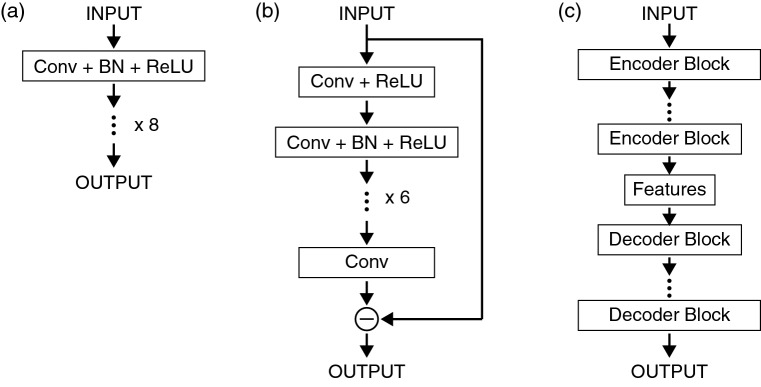
Network architectures of pCNN (**a**), DnCNN (**b**), and CAE (**c**). *Conv* : one-dimensional convolution, *BN* : batch normalization, *ReLU* : rectified linear unit.

Figure 1 illustrates the NR intensity profiles simulated for a 100-nm-thick film with the scattering length density (SLD) of $7.1 \times 10^{-4}$ $\hbox {nm}^{-2}$ (equivalent to that of deutertated PMMA) at an incidence angle of 0.3$^\circ$ with the total neutron numbers of 50,000, 10,000, 2500, and 1000, which correspond to 100%, 20%, 5%, and 2% of the standard measurement condition, respectively. The NR intensity profiles are shown as the neutron count against the momentum transfer, $q = 4\pi \sin \theta /\lambda$, where $\theta$ and $\lambda$ are the incident angle and wavelength of the neutron beam, respectively. The green curve and blue filled circles in each panel indicates the theoretically calculated ground truth profile and simulated NR data, respectively. This indicates that the statistical noise increases with a decrease in the acquired number of the neutrons. The oscillation pattern, so-called the Kiessig fringes, at *q* higher than 0.15 $\hbox {nm}^{-1}$ is an important spectral information for analyzing the NR data; however, it is not clearly observed for the data with the neutron counts of 2500 and 1000.

**Figure 3:**
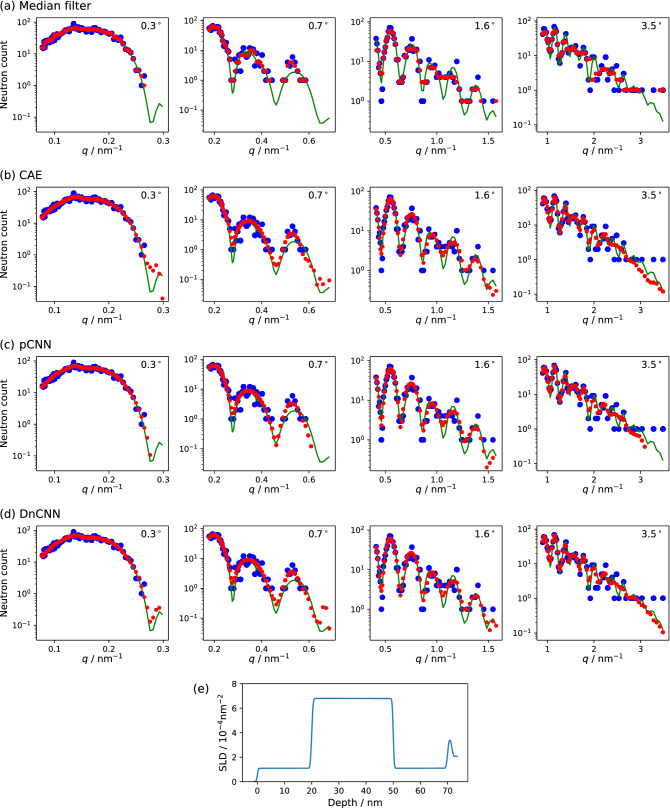
Predicted NR intensity profiles (red circles) by median filter (**a**), CAE (**b**), pCNN (**c**), and DnCNN (**d**) from the data with 5% neutron counts of the standard condition for the SLD distribution shown in (**e**). The green curves and blue circles represent the ground truth and simulated data, respectively.

Supervised training was carried out using CNNs for a data set of the simulated NR and the ground truth data. A plain CNN (pCNN) shown in Fig. 2a was used as a neural network model for the training, which comprises eight layers of one-dimensional convolution, batch normalization, and rectified linear unit. Recently, a denoising CNN (DnCNN) has been proposed as a powerful network model for the image noise reduction, which is designed to predict the difference between the noisy and clean images^20^. In this study, the DnCNN was modified for the one-dimensional data of NR as shown in Fig. 2b and it was used for the training. The training based on the convolutional autoencoder (CAE, Fig. 2c) was also conducted. The details on the network architecture is described in the Supplementary Information. Figure 3a–d show the results for the simulated NR data with the acquisition neutron counts of 5% of the standard condition for a sample structure with the SLD distribution shown in the panel (e). The blue filled circles, green curve, and red circles in each panel represent the simulated raw data, ground truth, and predicted data by the denoise processes, respectively. Figure 3a shows the result of a one-dimensional median filter with a window size of 5, which is a non-linear digital filter used for the noise reduction of the signals and images in many applications. Whereas it appears that the statistical noise for the data at 0.3$^\circ$ was reduced, the fringe pattern for the data at 1.6$^\circ$ and 3.5$^\circ$ is not reproduced. Thus, the NR data with such a low signal intensity cannot be used for the structure analysis even though it is processed by the median filter. The results using the CNN models are shown in Fig. 3b–d, and they clearly indicate that all data predicted by the deep learning show a much better fit to the ground truth compared to the median filtered result. The predicted profiles by CAE, pCNN, and DnCNN appear similar to each other; however, a significant difference can be observed at the high *q* end for each incident angle. The predicted profiles by the pCNN do not have the data points for $q > 0.26$ $\hbox {nm}^{-1}$ at 0.3$^\circ$, $q > 0.6$ $\hbox {nm}^{-1}$ at 0.7$^\circ$, and $q > 3$ $\hbox {nm}^{-1}$ at 3.5$^\circ$, indicating that the pCNN has difficulty in predicting the NR profile from the weak intensity less than the a neutron count less than 1. On the other hand, the CAE and DnCNN can provide the results at the above *q* region; however, the latter yields a better fit to the ground truth. The raw and predicted NR profiles for the other structures are shown in Supplementary Figs. S4–6 of the Supplementary Information.

Here, we discuss the uncertainty of the NR profile predicted by CNN. In deep learning, the analytical relation between the input and predicted NR data is hidden. Therefore, the uncertainty for the predicated NR profile by DnCNN was evaluated as the standard deviation for the predicted NR profiles from simulated data repeatedly for the same structure shown in Fig. 3e (the details are described in the Supplementary Information). Supplementary Figure S7 in the Supplementary Information shows the simulated NR data and the predicated data by DnCNN with the error bar. The uncertainty for the reflectivity for the predicated data was smaller than that for the simulated NR data. The averaged ratio of the standard deviation of the predicted reflectivity to that of the simulated data was evaluated to be 0.404, indicating that the uncertainty was reduced by ca. 60% by the prediction of the NR data using DnCNN.

As quantitative measures to discuss the difference between the ground truth and predicted data, we introduce the peak signal-to-noise ratio (PSNR) and structure similarity index measure (SSIM) referred to the ground truth data. A higher value of PSNR indicates the lower absolute error from the ground truth data. The PSNR for the data shown in Fig. 3 are 23.5, 27.5, 27.6, and 28.0 dB for the prediction by the median filter, CAE, pCNN, and DnCNN, respectively. The SSIM indicates the similarity between the two data and provides a value of unity for the exactly matched data. For the NR data, a higher value of SSIM means that the spectral information such as peaks and fringes is less distorted from the ground truth. The SSIM in Fig. 3 are 0.710, 0.878, 0.875, and 0.888 for the prediction by the median filter, CAE, pCNN, and DnCNN, respectively. Both the PSNR and SSIM for the predicted data obtained using the CNN models are considerably higher than those for the median filtered data.

**Table 1:** PSNR and SSIM for the raw and predicted NR data.

Acquisition number		Prediction model				
		Raw	Median	CAE	pCNN	DnCNN
100%	PSNR/dB	29.8	–	–	—	–
	SSIM	0.937	–	–	—	–
20%	PSNR/dB	27.4	27.3	31.8	30.4	31.7
	SSIM	0.813	0.775	0.909	0.911	0.918
10%	PSNR/dB	24.9	25.8	30.6	30.1	31.1
	SSIM	0.725	0.724	0.877	0.886	0.894
5%	PSNR/dB	22.6	24.0	28.7	28.1	29.3
	SSIM	0.627	0.664	0.826	0.833	0.845
2.5%	PSNR/dB	20.6	22.3	26.9	26.6	27.4
	SSIM	0.521	0.587	0.765	0.758	0.785
1.25%	PSNR/dB	18.7	20.6	25.1	24.6	25.5
	SSIM	0.407	0.487	0.696	0.658	0.715

In order to discuss the effect of the acquisition number of the signal neutron, we generated the simulated NR profiles for the random SLD structures of 2000 separately from the training and validation data sets with the neutron numbers of 20%, 10%, 5%, 2.5%, and 1.25% of the standard condition. For each acquired neutron number, the averaged values of PSNR and SSIM were evaluated over the 2000 structures. Table 1 summarizes the PSNR and SSIM of the raw and predicted data obtained using the neural network models. The data for the standard condition and the result of the one-dimensional median filter are also indicated therein. For the raw data, the PSNR and SSIM decrease with a decrease in the detected neutron, indicating that the information about the spectral shape is lost with an increase in the shot noise. The median filter is effective in terms of the PSNR for the NR data with small neutron counts less than 10% of the standard condition. It improves the PSNR whereas it does not largely affect the SSIM compared to the raw data. This indicates that the median filter reduces the noise for the low-count NR data without a significant loss of the spectral information. The digital filtering method cannot extract the spectral information hidden in the large statistical noise. Moreover, the maximum noise reduction is less than 2 dB, and therefore, the effect of the digital filtering for the NR data remains limited. The data processing based on the deep learning shows an increase in the PSNR by several dB in the most cases, indicating a considerably better noise reduction compared to the median filter. For the NR data with a 5% acquisition number, the predication using DnCNN improved the PSNR from 22.6 to 29.3 dB, and this is comparable to the raw data acquired in the standard condition. This suggests that the noise reduction with the deep learning technique enables the structural analysis of the NR data with the 20-fold smaller signal intensity compared to that under the standard condition. The SSIM for the data predicted by the CNN shows a higher value compared to the raw data, indicating that a spectral feature such as the fringe pattern hidden in the raw data can be recovered by the current data processing technique. This signal recovery is exemplified in Fig. 3. The peak and valley structure of the Kiessig fringe at incidence angles of 0.7, 1.6, and 3.5$^\circ$ are not viewed in the raw or median-filtered data. On the other hand, the fringe pattern is reproduced clearly in the predicted profiles (the red circles in Fig. 3b–d). The significant dependence of the PSNR and SSIM on the neural networks was not observed for the network models employed here. However, the predicted results of the CAE, pCNN, and DnCNN were slightly different from each other as discussed above; therefore, the further improvement can be expected by optimizing the neural network structure. Thus, the deep-learning-based data processing for NR reduces the noise significantly and reproduces the ground truth signal from the data with a large statistical error.

**Figure 4:**
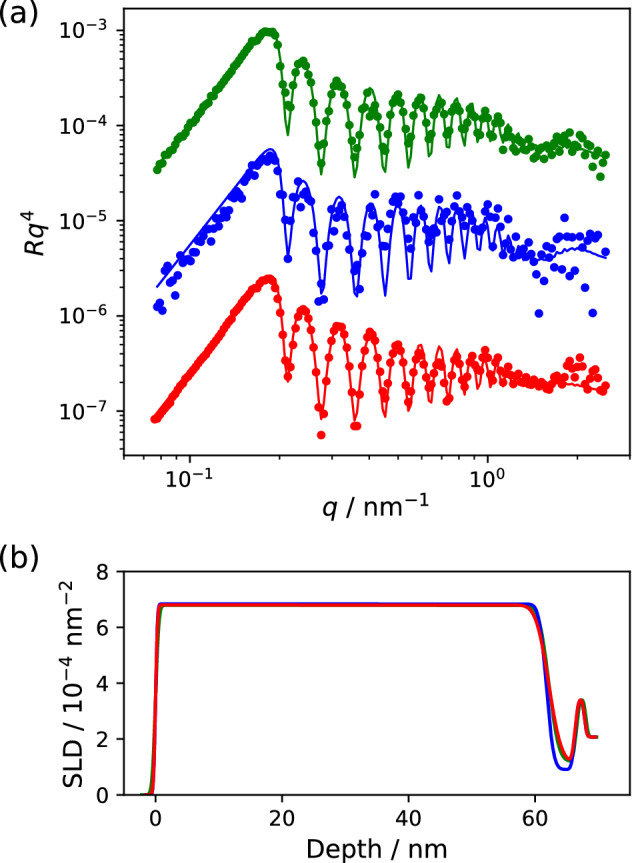
Neutron reflectivity profiles (**a**) and depth distribution of SLD obtained by the fitting analysis (**b**). The standard, low-count, and predicted data in each panel are colored in green, blue, and red, respectively. The circles and solid curves in (**a**) indicate the experimental data and fitted results, respectively. The reflectivity data are vertically shifted for visibility.

**Table 2:** Fitting results for the raw and predicted NR data.

		Standard	Low-count	Predicted
Layer 1	SLD/10$^{-4}$ $\hbox {nm}^{-2}$	$6.79 \pm 0.011$	$6.84 \pm 0.090$	$6.80 \pm 0.059$
	Thickness/nm	$62.2 \pm 0.034$	$61.8 \pm 0.186$	$62.1 \pm 0.228$
	Roughness/nm	$0.42 \pm 0.013$	$0.234 \pm 0.185$	$0.243 \pm 0.076$
Layer 2	SLD / 10$^{-4}$ $\hbox {nm}^{-2}$	$1.19 \pm 0.010$	$0.905 \pm 0.493$	$1.13 \pm 0.059$
	Thickness/nm	$4.35 \pm 0.087$	$4.51 \pm 0.292$	$4.15 \pm 0.055$
	Roughness/nm	$1.22 \pm 0.049$	$0.904 \pm 0.264$	$1.57 \pm 0.275$
Silicon oxide$^a$	SLD / 10$^{-4}$ $\hbox {nm}^{-2}$	3.47	3.47	3.47
	Thickness/nm	1.60	1.60	1.60
	Roughness/nm	0.40	0.40	0.40
Substrate$^a$	SLD / 10$^{-4}$ $\hbox {nm}^{-2}$	2.07	2.07	2.07
	Thickness/nm	$\infty$	$\infty$	$\infty$
	Roughness/nm	0.30	0.30	0.30

$^a$Fixed values in the fitting procedure.

The denoise model for NR was applied to the experimental data for a multi-layered PMMA film, which was prepared by the deposition of a 60-nm-thick film of deuterated PMMA (d-PMMA) onto a 4-nm-thick hydrogenated PMMA (h-PMMA) film formed on a silicon wafer. The green circles in Fig. 4a show the NR profile obtained by a reflectometer SHARAKU at a standard condition (with the acquired neutron counts of 50015, 20121, 20052, and 20507) at the incident angles of 0.3$^\circ$, 0.7$^\circ$, 1.6$^\circ$, and 3.5$^\circ$, respectively. Each NR data is normalized by the incident neutron beam to obtain the reflectivity, *R*, and multiplied by $q^4$ at the *q*-range of 0.07–2.5 $\hbox {nm}^{-1}$. The blue circles represent the NR data for the identical sample with the 5% acquired neutrons (ca. 2500 at the incidence of 0.3$^\circ$ and ca. 1000 at the other angles), indicating the NR profile with large noise compared to the data acquired at the standard condition. The red circles represent the predicted NR profile by the DnCNN model from the data with the 5% neutrons. This indicates that the predicted NR looks almost the same as the data observed at the standard condition. Each NR profile was fitted to a two-layered film model on a silicon wafer. The fitting analysis was conducted by the differential evolution algorithm and the uncertainty of the best-fitted parameter was calculated by the Hessian matrix^30^. The solid curves in Fig. 4a,b show the fitted NR profile and the SLD distribution obtained for the standard data (green curve), low-count data (blue), and predicted data (red). Although the obtained SLD profile by the low-count NR data was different from that obtained for the standard data at the depth of 60–70 nm as shown in Fig. 4b, the result of the fitting analysis of the predicted data was in good agreement with the standard data. The obtained best fitted parameters with the standard deviation are summarized in Table 2. The values of the thickness and the SLD of the layer 1, which corresponds to the d-PMMA layer, are almost the same for all of the data. These structural parameters are mainly characterized by the critical *q* of the total reflection and frequency of the Kiessig fringe pattern^2^, which are clearly observed also in the NR profile with 5% neutron counts (the blue circles in Fig. 4a). Therefore, the thickness and SLD of the d-PMMA layer can be determined from the NR data with an accuracy less than a few percents. On the other hand, the discrepancy at the depth of 60–70 nm for the standard and low-count NR data results from the inaccurate fitting for the h-PMMA layer (layer 2). The reflection interference related to the thin h-PMMA layer appears at a $q > 1$ $\hbox {nm}^{-1}$ of the NR profile. The large statistical error caused by the low reflectivity at such a high *q* range resulted in an inaccurate evaluation with a large uncertainty of the SLD and thickness for the h-PMMA layer from the low-count data. On the other hand, the DnCNN-based data processing predict the NR profile with small error, resulting in a fitting result with a relatively low uncertainty. The analysis of the predicted data and the standard data provided almost the same results as shown in the SLD profiles in Fig. 4b and Table 2. However, some parameters, such as the thickness and roughness of the layer 2, show a difference beyond the uncertainty. This could be caused by the distortion introduced by the neural network. In Fig. 4a, the predicted data (red circles) shows the decrease in the peak intensity only at $q = 0.7$ $\hbox {nm}^{-1}$ in the Kiessig fringe pattern. Such the distortion of the NR profile at a certain *q* range is not reproduced in the fitting procedure, resulting in an error in the fitted results. Whereas the difference of the results is small for the current data, the potential effect of the distortion of the NR profile should be considered for the application of the data processing by the neural networks. Thus, the deep learning-assisted signal restoration from a noisy NR data provided almost the same analysis results as the standard data despite the twenty-fold difference in the acquired neutron signals. This indicates that the signal restoration method based on a neural network enables the structure analysis of the NR data with a weak signal by an order of magnitude compared to that for the conventional conditions, resulting in a substantial improvement of the acquisition time in the NR measurement.

**Figure 5:**
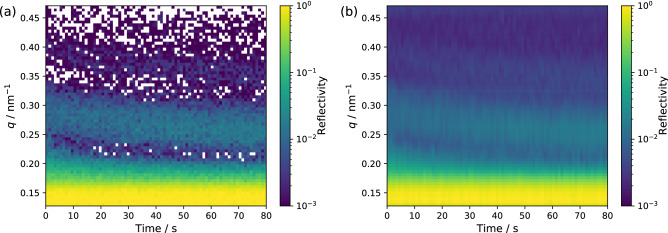
Raw (**a**) and deep learning-processed (**b**) time-dependent NR profiles for a 45-nm PVA film in heavy water. The neutron beam was incident from the substrate at an angle of 0.5$^\circ$. DnCNN was used as a neural network in data processing.

The remarkable reduction of the data acquisition time in NR has considerable importance in studying the dynamics of the surface/interface structure because it is directly related to the temporal resolution. The enhancement of the temporal resolution in the time-resolved NR measurement is demonstrated by the characterization of a structural change of a thin film of PVA. Figure 5a is the reflectivity map with *q* and time (*t*) axes showing the time-dependent NR profile for a 45-nm-thick film of PVA on a silicon wafer after the immersion in heavy water, which was observed with the time division of 1 s. The average neutron number for each time window is 2250. The PVA thin film absorbs the heavy water because of the high solubility of PVA in water; therefore, the low-*q* shift of the peak and valley positions in the Kiessig fringe pattern is observed because of an increase in the total thickness caused by the swelling of PVA with the heavy water. Such the time dependence of the NR profile is seen in Fig. 5a. However, each profile cannot be used for the fitting analysis to examine the thickness and SLD of the sample film because of the existence of a lot of data points without the signal (white points in Fig. 5a). On the other hand, the statistical noise was removed by the DnCNN network as shown in Fig. 5b, indicating that the deep learning denoise processing enhances the effective temporal resolution in time-resolved NR measurements. Although the noise in the *q*-axis was removed by the CNN-based data processing technique, the noise in the *t*-axis still remains in Fig. 5b. This is because the data processing by DnCNN was independently applied to each time-sliced NR profile without considering the correlation in the *t*-axis. The neural network considering not only the *q*-dependence but also the *t*-dependence of the reflectivity would produce further improvement in the data analysis of the time-dependent experiments. Whereas the temporal resolution is limited to on the order of minutes in the conventional NR, the current method allows discussing the structure change on the order of seconds.

## Conclusion

A novel data processing method was introduced for NR. Deep learning using convolutional neural networks allows restoring the NR profile from the data with extremely large statistical noise. A quantitative comparison among the NR profiles with various acquired neutron numbers showed that the deep learning approach allows improving the statistical noise by one order of magnitude. The application of the current technique to the experimentally obtained data with a 20-fold smaller signal compared to the conventional acquisition condition provided a structure analysis result with the same accuracy as the conventional data. This method achieves a remarkable reduction of data acquisition time of the NR and a higher resolution in the time resolved measurement to examine the time evolution of the surface/interface structure in in situ and operando conditions. Moreover, a measurement time as long as a few days for a tomographic technique for spatially resolved imaging can be reduced to the order of several hours. Such an improvement would also be attained by increasing the beam power; however, the construction of the neutron source with a higher intensity by an order of magnitude has technical and financial difficulties. Therefore, the current approach in NR based on the deep learning would significantly contribute to the development of temporally/spatially resolved NR method. This method helps not only shorten the acquisition time but also minimize the effect of the radiation dose on a specimen. Although the deep learning training here presumed the NR experiment with SHARAKU installed at MLF, J-PARC, this method can be employed for other instruments by deep learning training using the data simulated considering the measurement condition for each of them. Moreover, this approach is applicable not only to NR but also to other experimental methods such as small angle scattering and spectroscopy using neutron, X-ray, and light. This technique can open up a new horizon in the structural analysis of the surface and interface of materials in various research fields.

## Supplementary Information

Supplementary Information 1.

Supplementary Information 2.

## Data Availability

The sample python scripts with the data sets are available as the Supplementary Information. The experimental data used in the current study are available from the corresponding author on reasonable request.
