# Supplementary material for: Deep learning approach for an interface structure analysis with a large statistical noise in neutron reflectometry
Source: Sci Rep. 2021 Nov 22;11:22711. doi: 10.1038/s41598-021-02085-6 (PMC8608885; doi:10.1038/s41598-021-02085-6)
Supplement: Supplementary file 1 — Supplementary Information 1. [file 41598_2021_2085_MOESM1_ESM.pdf]

Supporting Information

Deep learning approach for an interface structure analysis with a large statistical noise in neutron reflectometry

Hiroyuki Aoki[1,2], Yuwei Liu[2], and Takashi Yamashita[3]

[1]Materials and Life Science Division, J-PARC Center, Japan Atomic Energy Agency
[2]Institute of Materials Structure Science, High Energy Accelerator Research Organization
[3]AdvanceSoft, Co. Ltd.

1 Simulated neutron reflection profile

For the supervised deep leaning, the simulated NR data was used as the training data. The neutron intensity profiles for the computer-generated structures were simulated considering the measurement condition of a neutron reflectometer, SHARAKU, installed at Materials and Life Science Experiment Facility (MLF) in Japan Proton Accelerator Research Complex (J-PARC).

A neutron reflectometry (NR) profile is characterized by the depth distribution of the neutron scattering length density (SLD). The sample structure was defined as stacked layers on a substrate with SLD of 2.07×10^{-4} nm^{-2} (equivalent to silicon). The sample structures with the layer number from 1 to 16 were generated. The SLD and thickness of each layer was randomly determined in the ranges of $0.5 \times 10^{-4} - 7.5 \times 10^{-4}$ nm^{-2} and 1 – 100 nm, respectively. The roughness for each interface was a constant value of 0.3 nm. In addition to the randomly generated stacked sample structures, the sample consisting of repeated bilayers were also generated. Randomly generated two layers with the SLD of $0.5 \times 10^{-4} - 7.0 \times 10^{-4}$ nm^{-2} and thickness of 1 – 10 nm was stacked on a substrate with SLD of 2.07×10^{-4} nm^{-2} repeatedly with the number of 2 – 16. In the structure generation process, we discarded the sample structure which has a layer where the difference of the SLD between the neighboring layers was

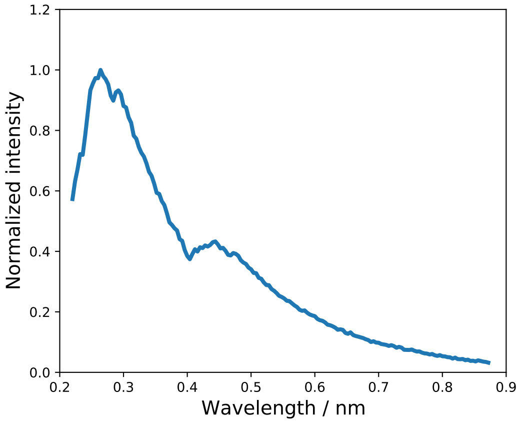

Figure S1. Spectrum of the incident neutron used for the NR experiment and simulation, which was observed by a neutron reflectometer SHARAKU at MLF of J-PARC.

less than 0.1×10^{-4} nm^{-2} or the total thickness of which is more than 160 nm. For the deep learning training, the total number of the generated structures was 2.4×10^5. The NR profiles with limited numbers of acquired neutrons were simulated for the ground truth NR profiles. The process of the generation of the neutron reflection intensity profile for each structure is as follows. (1) The theoretical reflectivity against the momentum transfer, q, was calculated, which is defined as $q = 4\pi \sin\theta/\lambda$ by the incident angle θ and the wavelength λ of neutron. (2) The reflection intensity profiles at incident angles of 0.3, 0.7, 1.6, and 3.5° were calculated by the theoretical reflectivity and the wavelength distribution of the neutron intensity at SHARAKU shown in Figure S1. (3) The neutron events were generated to reproduce the neutron reflection intensity profiles against q with the total neutron numbers of 10%, 5%, 2.5%, and 1.5% of the standard condition (the number of detected neutrons of 50000 for the incident angle of 0.3° and 20000 for the other angles). The events were generated according to the Poisson statistics, because the statistics of the acquired neutrons at SHARAKU is in good agreement with the Poisson distribution as shown in Figure S2. Thus, the total number of the training data sets was 9.6×10^5.

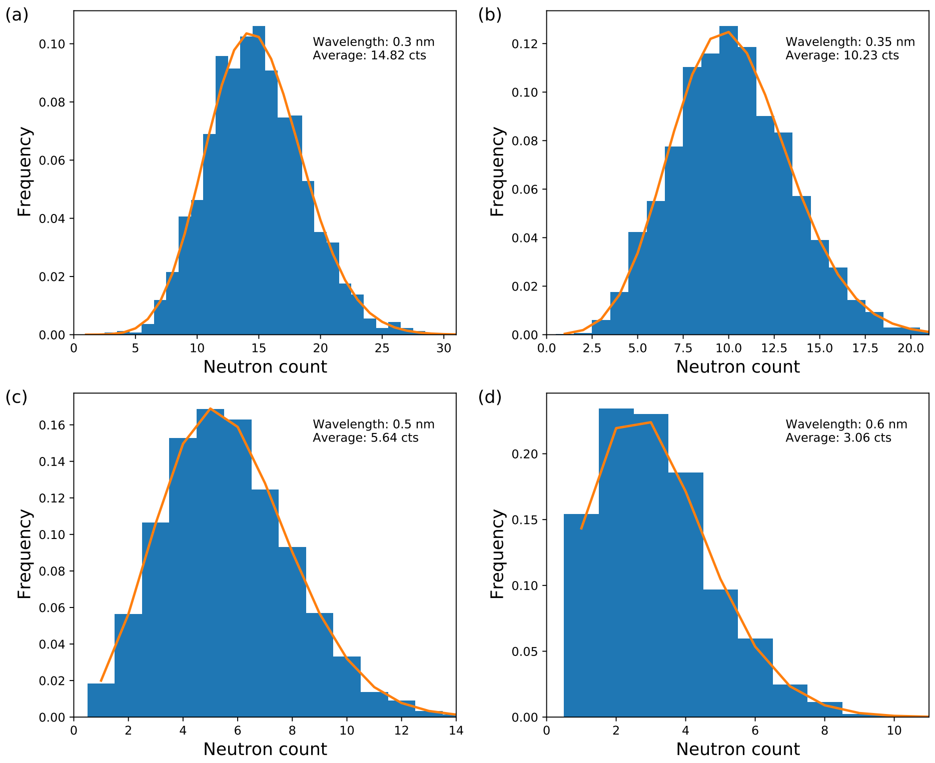

Figure S2. Histograms of the experimentally detected neutrons in 0.2-sec time bin for the incident beam (blue bars) and Poisson distributions (orange curves) at wavelengths of 0.3 (a), 0.35 (b), 0.5 (c), and 0.6 nm (d). The Poisson distribution in each panel was calculated to give the average value determined by the experiment.

2 Deep learning

The supervised training by the network architectures of plain convolutional network (pCNN), denoising CNN (DnCNN), and convolutional autoencoder (CAE) was conducted for the ground truth and simulated NR profiles. The network structure of pCNN is similar to a typical convolutional neural network architecture widely used for various tasks and it consists of 8 layers of a block including one-dimensional convolution (Conv), batch normalization (BN), and rectified linear unit (ReLU). The original DnCNN model proposed to apply BN and residual learning to improve the efficiency of the learning process and to improve the denoising performance. The main difference between typical CNNs used for noise reduction and original DnCNN is that a typical CNN removes the noise from the input data, while DnCNN infers the noise components in the input data. In this study, the DnCNN model was modified to process the one-dimensional data and to output the denoised NR profile by subtracting the output of the last layer from the input data. The CAE is divided into the encoder and decoder parts. network for the encoder blocks and decoder blocks. In the current model, the encoder consists of 3 blocks of Conv, pooling layer, and ReLU. The decoder consists of 3 decoding blocks. Each block consists of an up-projection with linear interpolation, convolutional processes with Conv and BN, and ReLU. The decoder compute the final output from the compressed representation of input data by the encoder. We used the mean squared error (MSE) as the loss function and Adam optimizer as the optimization function to train the models, with a learning rate of 10^{-3} and an epoch number of 128. In updating the model parameters, the plain CNN model and CAE models evaluated the difference between the model output and the ground truth, while the DnCNN models evaluated the difference between the model output and the noise component of the input data which is obtained by subtracting the ground truth from the simulated NR. The training was carried out using Pytorch on a workstation equipped with a CPU of Core i9, Intel, memory of 128 GB, and two GPU boards of GeForce RTX3090, NVIDIA. In the training procedure, 80% and 20% of the data sets were used for the training and the validation, respectively.

3 NR experiments

In order to discuss the applicability of the deep learning data processing to the experimental data, the NR measurements were conducted for thin films of poly(methyl methacrylate) (PMMA) and poly(vinyl alcohol) (PVA).

The PMMA sample consisted of deuterated PMMA with the weight-average molecular weight (M_w) of 1.04×10^4 (d-PMMA) and hydrogenated PMMA with M_w of 1.06×10^5 (h-PMMA). A 100-nm thin film of h-PMMA was prepared by spin-casting from a toluene solution on a silicon wafer and annealed at 150°C for 48 h. Repeated sonication treatments in toluene resulted in a 4-nm-thick film of h-PMMA. A 60-nm-thick film of d-PMMA was prepared on another wafer. It was floated on a water surface and scooped onto the 4-nm-thick h-PMMA film to prepare a stacked film of h-PMMA and d-PMMA. The thickness of the h-PMMA and d-PMMA films at the sample preparation process was observed by spectroscopic ellipsometer measurements (M-2000, J.A. Woollam Co. Inc.). The NR measurement of the PMMA film at SHARAKU was conducted at the incidence angles of 0.3, 0.7, 1.6, and 3.5°.

The PVA sample with a thickness of 45 nm was prepared by spin-coating a aqueous solution

of PVA (the degree of polymerization of 1800, Wako Pure Chemicals, Inc.) on a silicon wafer. The sample film was set to a measurement cell shown in Figure S3, which is equipped with a water reservoir. In the NR measurement of the PVA thin film, the neutron beam was incident from the substrate at an angle of 0.5°. After starting the data acquisition for the PVA film in an atmosphere, heavy water (D_2O 99.9%, Cambridge Isotope Laboratories, Inc.) was injected to immerse the sample film by opening the solenoid valve of the reservoir. The measurement was carried out for 60 min after the immersion to obtain the NR profile dependent on the elapsed time with a time window of 1 s.

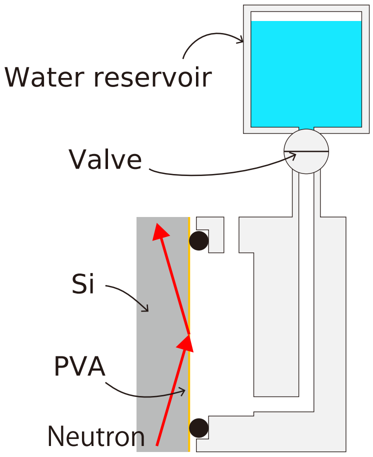

Figure S3. Sample cell for time resolved NR measurement of the structure change in water.

4 Predicted NR for various structures

Figures S4–S6 show the examples of the predicted NR intensity profiles by median filter (a), CAE (b), plain CNN (c), and DnCNN (d) from the simulated data with the neutron numbers of 2500 for the incidence angle of 0.3° and 1000 for the other angles (5% neutron counts of the standard condition). The sample structures shown here were not used for the training or validation in the deep leaning process.

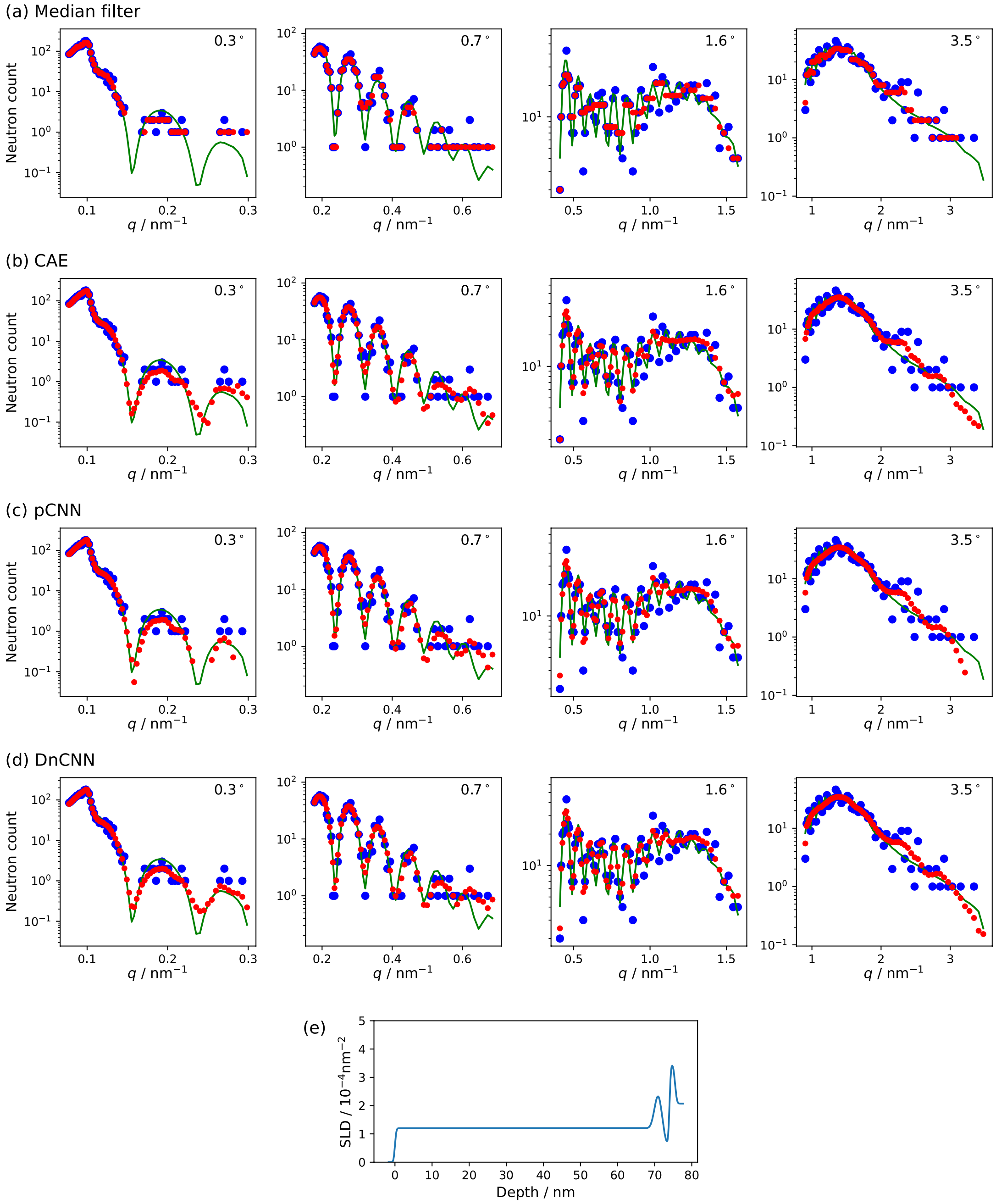

Figure S4. Predicted NR intensity profiles by median filter (a), CAE (b), plain CNN (c), and DnCNN (d) for the SLD distribution shown in (e). The green curves, blue circles, and red circles indicate the ground truth, generated data, and predicted results, respectively. (a) PSNR: 25.1 dB, SSIM: 0.602. (b) PSNR: 28.9 dB, SSIM: 0.812. (c) PSNR: 29.3 dB, SSIM: 0.825. (d) PSNR: 29.9 dB, SSIM: 0.849. The green curves, blue circles, and red circles indicate the ground truth, generated data, and predicted results, respectively.

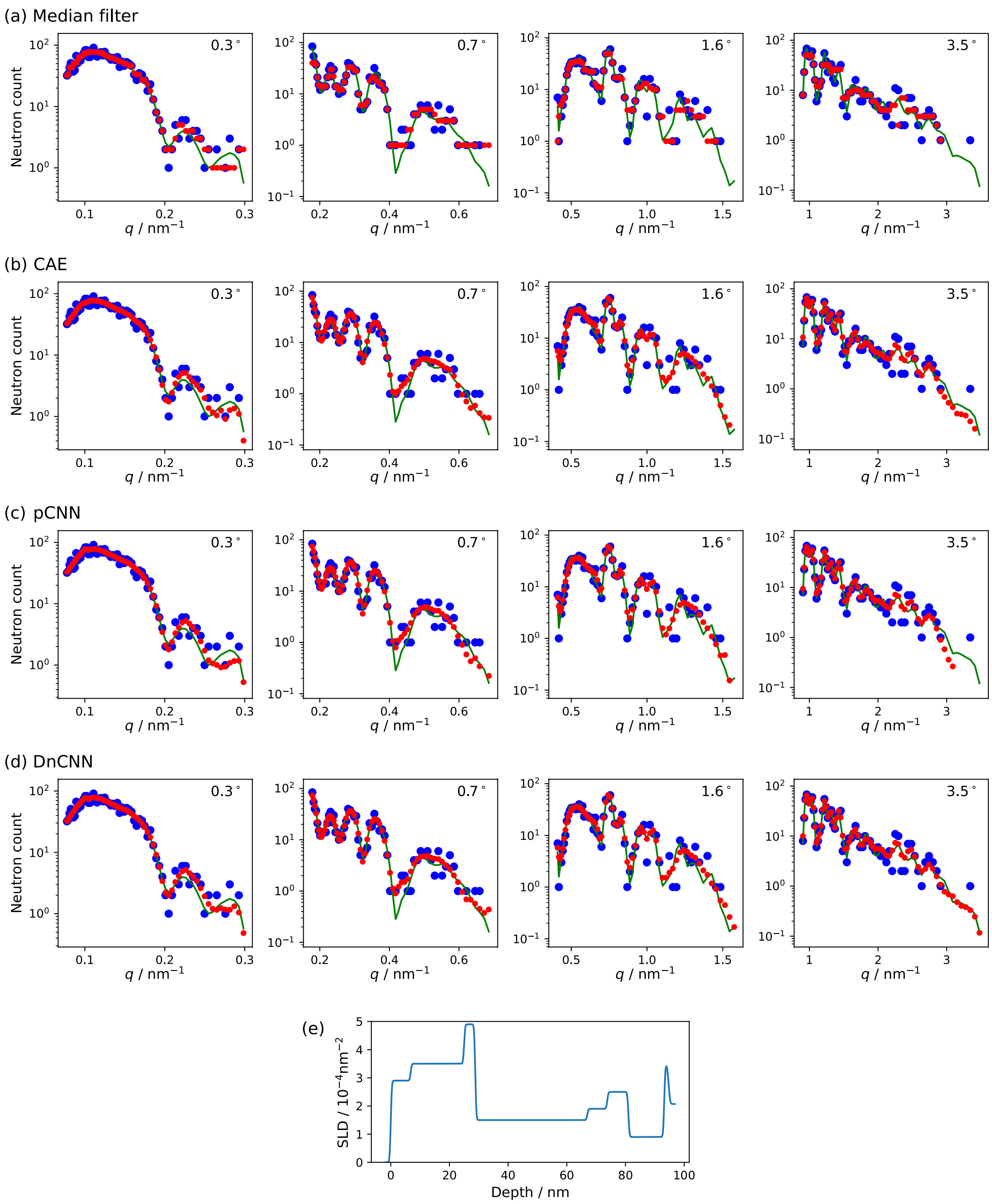

Figure S5. Predicted NR intensity profiles by median filter (a), CAE (b), plain CNN (c), and DnCNN (d) for the SLD distribution shown in (e). The green curves, blue circles, and red circles indicate the ground truth, generated data, and predicted results, respectively. (a) PSNR: 24.4 dB, SSIM: 0.711. (b) PSNR: 28.7 dB, SSIM: 0.876. (c) PSNR: 28.5 dB, SSIM: 0.878. (d) PSNR: 29.1 dB, SSIM: 0.889. The green curves, blue circles, and red circles indicate the ground truth, generated data, and predicted results, respectively.

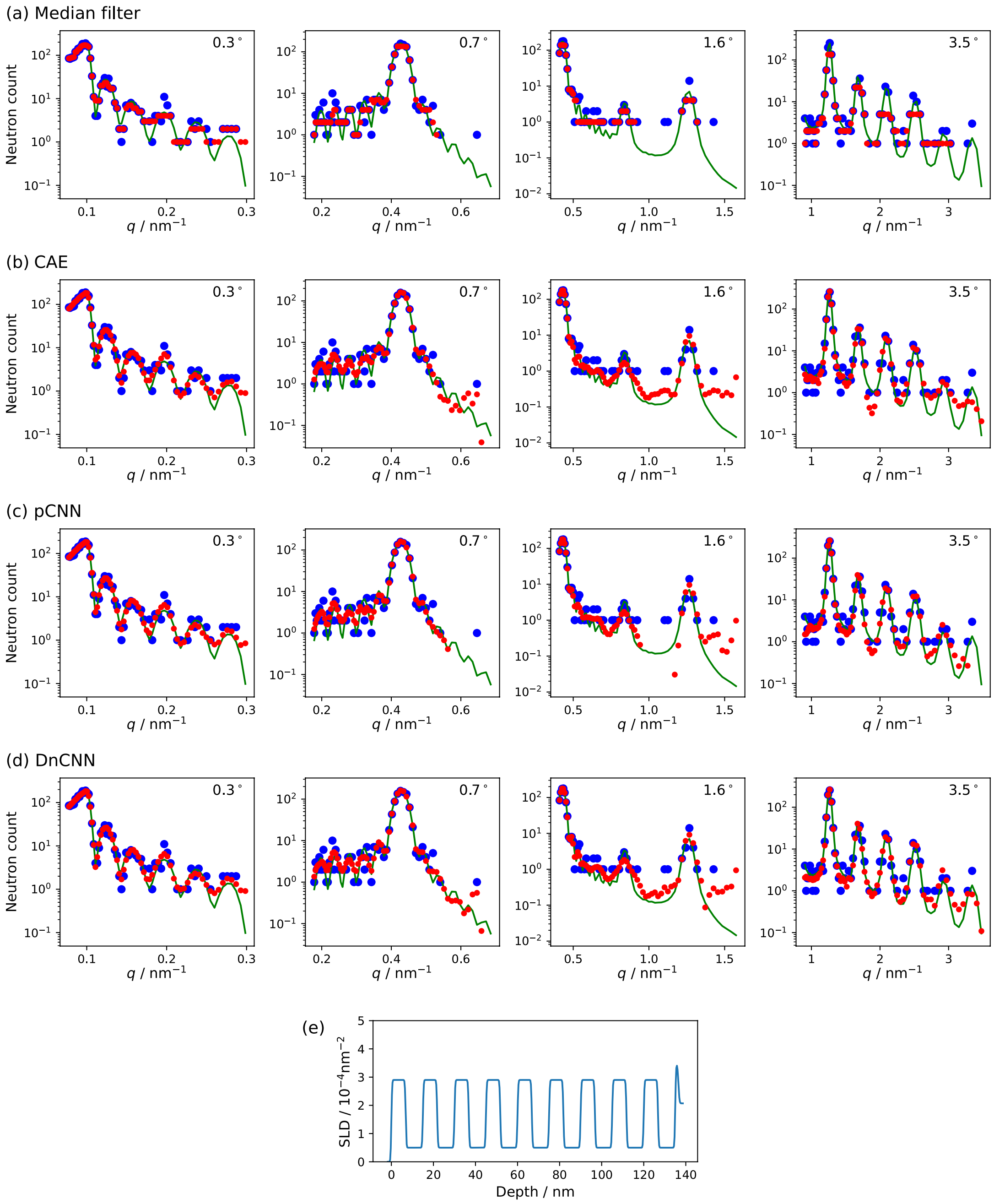

Figure S6. Predicted NR intensity profiles by median filter (a), CAE (b), plain CNN (c), and DnCNN (d) for the SLD distribution shown in (e). The green curves, blue circles, and red circles indicate the ground truth, generated data, and predicted results, respectively. (a) PSNR: 24.0 dB, SSIM: 0.640. (b) PSNR: 28.4 dB, SSIM: 0.806. (c) PSNR: 28.3 dB, SSIM: 0.783. (d) PSNR: 29.1 dB, SSIM: 0.836. The green curves, blue circles, and red circles indicate the ground truth, generated data, and predicted results, respectively.

5 Error propagation in NR profile

For the same structure shown in Figure 3b, 1000 NR profiles were generated with the 5% neutron count of the standard condition and processed by prediction of the true NR profile by the DnCNN. The simulation of the NR profile was performed for the incidence angles of 0.3°, 0.7°, 1.6°, and 3.5°. The uncertainty of the reflection signal for the predicted NR profile was evaluated as the standard deviation of the 1000 profiles. The uncertainty for the simulated data corresponds to the square-root of the neutron count. The simulated and predicted NR profiles are represented in Figure S7 with the uncertainty for each data point. In each panel, the figures at the left and right hand sides show the simulated and predicted NR profiles, respectively. The average of the ratio of the standard deviation for the predicated NR data to that for the simulated data was evaluated to be 0.404.

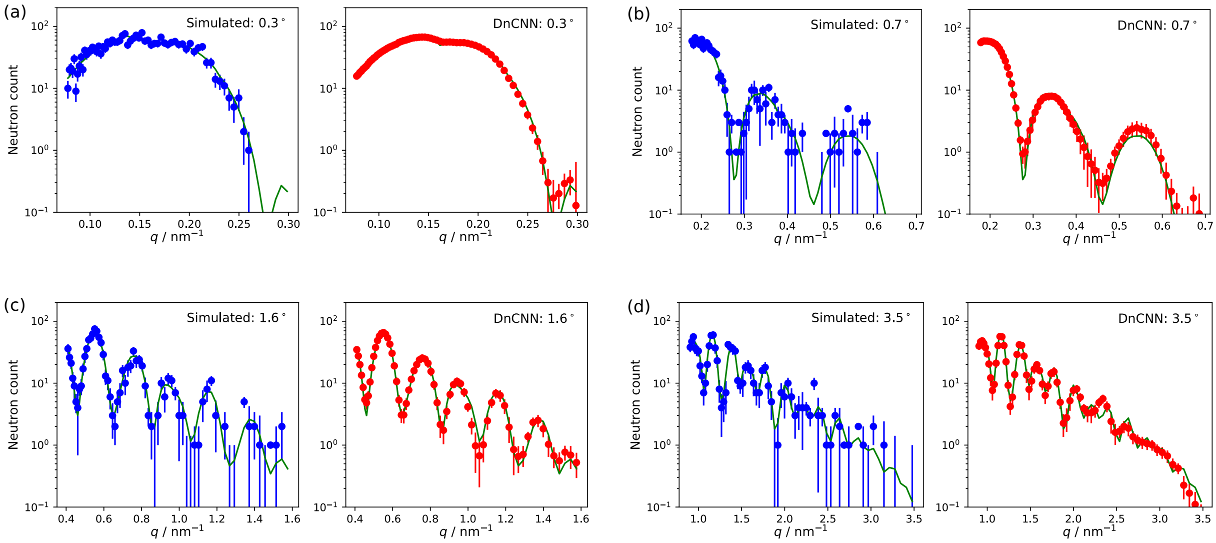

Figure S7. Simulated and predicted NR profiles with the uncertainty at the incidence angles of 0.3° (a), 0.7° (b), 1.6° (c), and 3.5° (d). The green curve in each figure indicates the ground truth NR profile.

6 Sample script

The sample python scripts for training and prediction of NR profiles are available in a ZIP archive with the data sets.
